# A systematic review exploring characteristics of youth with severe and enduring mental health problems (SEMHP)

**DOI:** 10.1007/s00787-023-02216-6

**Published:** 2023-04-24

**Authors:** C. H. Bansema, R. R. J. M. Vermeiren, R. de Soet, H. van Ewijk, L. Nijland, L. A. Nooteboom

**Affiliations:** 1 LUMC Curium-Department of Child and Adolescent Psychiatry, Leiden University Medical Center, Post Box 15, 2300 AA Leiden, The Netherlands; 2 Youz, Parnassia Group, The Hague, The Netherlands

**Keywords:** Severe distress, Enduring mental health problems, Comorbidity, Youth, Adolescent psychiatry, Systematic review

## Abstract

A small group of youth and emerging adults deals with severe and enduring mental health problems (SEMHP). Current mental health care struggles to recognize and treat this group timely and adequately, leaving these youth between the cracks of the system. A first step to improve care for this group is to gain a deeper understanding of the characteristics of youth with SEMHP. Therefore, this study aimed at reviewing current literature about this target group and what is known so far about their characteristics. We included 39 studies with a focus on youth aged 12–25 years with SEMHP. After critical appraisal, a content analysis and in-depth thematic analysis were conducted. According to the included studies, youth with SEMHP were characterized by severe distress and recurrent comorbid mental health problems, with pervasive suicidality. Further, underlying trauma, family conflicts, peer rejection, deep feelings of hopelessness, and psychosocial malfunctioning characterized SEMHP. It was described that for youth with SEMHP a pervasive pattern of dysfunction in multiple domains is present leading to a detrimental impact. Subsequently, this pattern exerts a reciprocal influence on the mental health problems, causing a vicious circle further worsening SEMHP. Our findings emphasize the need for a holistic approach and to look beyond the traditional classification system in order to meet the needs of these youth with wide-ranging comorbid mental health problems.

**Supplementary Information:**

The online version contains supplementary material available at 10.1007/s00787-023-02216-6.

## Introduction

Attention is urgently needed for youth and emerging adults (referred to as youth in this paper), who fall between the cracks of current mental health care. While for most youth in mental health services, mental health problems are treatable and transient, a small group of youth experiences severe and enduring mental health problems (SEMHP). Severe and enduring mental health problems include socio-emotional, behavioral, and academic difficulties, often resulting in severe self-harm or suicidal attempts [1, 2]. For youth with SEMHP, the current mental health care all too often seems unsuitable [3]. Current mental health care tends to focus on classifications: the assignment of a mental disorder to a set of criteria that interferes with daily functioning. However, youth with SEMHP are often assigned to multiple classifications without adequate attention to the underlying mental health problems. These classifications do not provide information about the causes and are therefore limited in guiding the diagnostic process [4]. At present, we lack the means to recognize SEMHP youth timely and correctly. Therefore, an approach beyond the classifications is needed. A first step to improve care for this group, is to gain a deeper understanding of the characteristics of youth with SEMHP.

Growing evidence, supporting the need for a different approach, shows the presence of an underlying dimension in adults with severe and enduring mental disorders: a common vulnerability for psychopathology, the P-factor [5, 6]. In a pediatric sample, similar results were found for the younger age group [2]. According to the P-factor theory, mental health disorders are interconnected and caused by transdiagnostic genetical and environmental factors [7]. The P-factor theory implies that individuals with a diversity of severe and enduring symptoms, are likely to share the same underlying vulnerability. Various circumstances—for instance, (sexual) abuse, personal loss, poverty or being bullied- may trigger the development of (severe and enduring) mental health problems, subsequently resulting in a diversity of classifications. The current focus in mental health care on specific classifications [8], lacks focus on underlying psychopathology, personal characteristics and factors that trigger a set of symptoms [5]. Moreover, although clinical practice does describe youths’ problems as severe and enduring, it remains unclear how we define or evaluate this severity [9]. To better understand youth with SEMHP, it is needed to look beyond standard symptoms and further discover explanatory factors and characteristics related to severe and enduring.

For adults with severe and enduring problems, Delespaul et al. formulated a description that enables to recognize them, based on clear inclusion and exclusion criteria [10]. This description includes: (a) a psychiatric disorder requiring care; (b) with severe disabilities in social functioning (which may fluctuate); (c) where the disability is both cause and effect; (d) which is not transient; and (e) where coordinated care from professionals is required [10]. Although helpful, these criteria are at best only partly applicable for youth, as it is difficult to establish whether youth’ mental health problems are temporary or may vanish due to their maturation [11]. In order to formulate a description of SEMHP that fits youth, further research on their characteristics is crucial.

The aim of this study was to explore the characteristics of youth with SEMHP from current literature. Since little research has been done on this specific group, we choose to conduct a descriptive systematic review. Based on a content and thematic analysis, an overview of characteristics will be provided on three levels: (a) current descriptions of severe mental health problems and enduring mental health problems; (b) contributing factors to the development and continuation of SEMHP; c) the impact of SEMHP.

## Methods

This systematic review was performed following the recommendations of the Preferred Reporting Items for Systematic Reviews and Meta-Analyses (PRISMA). A research protocol was prospectively registered in the International Prospective Register of Systematic Reviews (Prospero) in 2021 (CRD42021239131). To identify and describe themes in this systematic review, we conducted a content- and thematic analysis, consisting of the following five steps: framing questions, identifying relevant work, assessing the quality of studies, summarizing the evidence, and interpreting the findings [12].

### Search strategy

The search strategy was developed in collaboration with a research librarian from the Leiden University Medical Center. Four databases (PubMed, PsycINFO, Web of Science and the Cochrane Library) were searched using the search terms presented in Appendix A. Search terms were related to the following concepts of interest: (a) youth, such as children, pediatrics, teenagers, adolescents and youth; (b) mental health problems, mental disorders, psychiatric disorders; (c) severe and enduring including their synonyms; (d) biopsychosocial factors; (e) impact. Keywords were generated for each of these concepts by examining terminology used in recent review papers in mental health problems literature [13, 14]. These key words were combined with MeSH terms from the PubMed and Cochrane databases and Subject Headings for the PsychINFO database. In addition, we performed a search by hand: checking the reference lists of the included studies.

### Inclusion and exclusion criteria

To be included, studies had to meet the following eligibility criteria:

- Studies focused on youth and emerging adults (youth) aged 12–25 years. Studies with a broader age range were included as long as the mean age of the participants fell between 12 and 25 years.
- Studies focused on the characteristics of youth with severe and enduring mental health problems (SEMHP). A “characteristic” was defined as a feature belonging typically to a person or their environment serving to identify them. The definition of severe and enduring in terms of mental health problems was based on a definition of severe psychiatric problems for adults established by Delespaul [10], serving as a starting point. Thus, severe mental health problems were defined as: (a) serious/severe interrelated mental health problems that; (b) necessitate care; (c) with severe disabilities in social functioning. And enduring mental health problems were defined as (a) not transient/structural/persistent; or (b) recurring.
- Studies were peer-reviewed, including qualitative, quantitative and mixed-method studies.
- Studies were published between 1992 and 2023 [15], in a peer-reviewed, English-language journal. Full-text had to be available.

Studies were excluded according to the following criteria:

- Studies were not peer-reviewed, including but not limited to: editorials and letters, graduate-level thesis, conference abstracts and notes.
- Studies focused on physical diseases (medical conditions) or medical treatment.
- Studies focused on youth with a specific single mental health problem or mental disorder (e.g. solely a gaming disorder).
- Studies focused on treatment without any description of the target group.
- Studies focused on specific non-western population (e.g. native Indians).

### Study selection

**Fig. 1:**
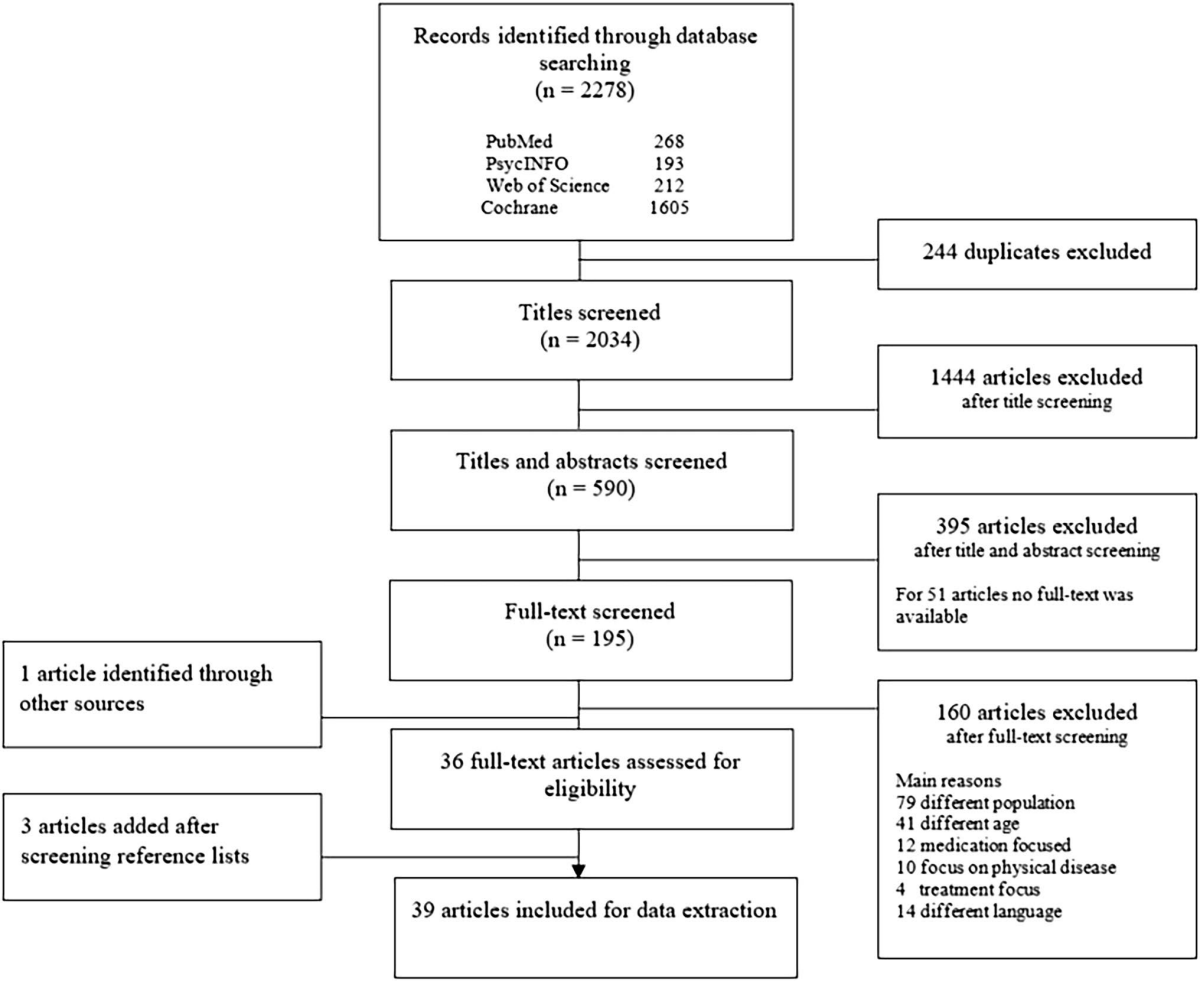
PRISMA flowchart showing the selection process

The initial database search returned 2034 published abstracts after removing duplicates. At the first stage, the main author (CB) screened the titles and excluded all studies concerning straightforward specific medical conditions. In the second stage, two researches (CB & RS) independently screened titles and abstracts and excluded studies based on the inclusion and exclusion criteria. Disagreements were discussed in order to reach consensus. If consensus could not be reached, a third researcher (HE) was consulted. An overview of the study selection process is presented in Fig. 1: PRISMA Flowchart [16].

### Data extraction and analysis approach

An extraction form, based on the Cochrane Data Extraction Template [17], was applied in the data extraction process. This form included study characteristics (i.e. research questions, study methodology, setting), source of evidence from eligible studies, and a description of the target population (i.e. type of mental health problems). In order to avoid publication bias, all studies were checked for using the same dataset. This was the case for eight studies, which were counted as one in the evaluation of evidence.

Subsequently, a combined thematic and content analysis was performed. The content analysis was conducted in order to quantify and examine the presence and meaning of themes [18]. In addition, the thematic analysis was conducted for identifying, examining and reporting themes within the data [19]. For both the thematic and content analysis, the same set of analytical interventions were operated as follows: preparation of the data (familiarizing), organizing the data (open coding, grouping codes), and synthesizing themes and reporting the results in categories [20]. The coding process was supervised by a second reviewer, experienced in qualitative literature reviews (LAN). For the thematic and content analysis, the results section of the included studies were coded. To code the data, a software program (Atlas.ti.9) was used. First, open coding was conducted in order to identify relevant text units. Also, selective coding was performed based on the biopsychosocial model [21]. Then, axial coding took place by grouping together similar codes using descriptive themes. We pre-defined the themes: Biological factors, Psychological factors and Sociological factors, based on the biopsychical model [21]. Also, we pre-defined the theme Descriptions and subthemes Severe and Enduring to gain more insight into the meaning of these terms. Then, to answer the research questions, all themes and subthemes were divided into the folowingthree pre-defined categories (a) the meaning of severe and enduring mental health problems (severe and enduring); (b) contributing factors to the development and continuation of SEMHP (contributing factors); and (c) the impact of youth experiencing SEMHP (impact). In order to prevent interpretation bias, a second reviewer (LAN) evaluated the identified themes on relevance and potential overlap.

### Quality assessment

The quality of the individual studies (case reports, case series, cross-sectional studies, qualitative studies, reviews, and cohort studies) was appraised using standardized checklists of the Joanna Briggs Institute (JBI) [22]. The Critical Appraisal Skills Program (CASP) was applied on randomized controlled trials [23], and case control studies [24]. The researchers used a predeveloped ranking system [25] in order to assess the study quality based on the checklist. The quality ranking system included the following three categories: high (more than 8 items checked), medium (6–8 items checked), and low (less than 6 items checked). An overview of the study characteristics and critical appraisal scores can be found in Appendix B.

### Strength of evidence

The strength of evidence of each subtheme was calculated [26], based on the following predefined criteria [25]:

- *Size of evidence*: the size of evidence was calculated using the number of studies within a subtheme. Subthemes consisting of 15 or more studies were graded as large (+); between 5 and 15 studies as medium (±); and less than 5 studies as small (−).
- *Quality of studies*: based on the critical appraisal checklists for individual studies the overall quality of the subtheme was assessed. High (+); was awarded to subthemes consisting of > 75% of studies appraised as high quality. Medium (±); was awarded to subthemes consisting of 25–75% of high quality studies. Low (−); was awarded to subthemes consisting of > 25% high quality studies.
- *Context*: the context of each subtheme was categorized into mixed or specific. Mixed (+) was assigned to subthemes consisting of studies including multiple contexts: comorbid psychiatric classifications with multiple mental health problems. Specific (−) was assigned to subthemes consisting of studies focusing on a specific context: a psychiatric classification with multiple mental health problems (e.g. eating disorder with suicidality).
- *Consistency*: subthemes including evidence pointing to similar conclusions were considered consistent (+); subthemes including studies on different subpopulations (youth with different psychiatric classifications, e.g. MDD with PTSD versus ED with suicidality), with inconsistent results were considered mixed (i.e. not consistent and not inconsistent, ±); and subthemes including studies directly countering findings based on the same subpopulation were considered inconsistent (−).
- *Perspective (source of evidence):* subthemes in which the source of evidence came from two or more perspectives (participants): youth, parents, professionals (e.g. practitioners) were considered mixed (+); and subthemes in which the source of evidence came from one perspective were considered single (−).
- *Area of life:* Subthemes with findings from different settings (e.g. in-patient and out-patient) were considered mixed (+); and subthemes with findings from one setting (e.g. household) were considered specific (−).

Based on the scores assigned in each subscale (i.e. size of evidence, quality of studies, context, consistency, perspective and area of life), the overall strength of evidence was calculated: very strong (++++), strong (+++), medium (++), limited (+), or no evidence (−).

## Results

### Study characteristics

This systematic review included 39 studies. Most studies were cross-sectional or cohort studies. Critical appraisal of individual studies resulted in 19 high quality studies, 18 medium quality studies, and two low quality studies. The included studies covered a variety of target group descriptions and classifications. An overview of all study characteristics can be found in Appendix B.

### Outcomes

The thematic analysis included three overarching categories, seven main themesand 20 subthemes (see Table 1 for an overview of categories, themes, and subthemes). The strength of evidence was evaluated for each subtheme based on the predeveloped rating scheme, with most subthemes being strong (*n* = 14) or medium (*n* = 9), and only one subtheme with limited to no evidence. A detailed description of the strength of evidence per subtheme can be found in Appendix C.

**Table 1:** Overview of categories, themes, and subthemes

Category	Main theme	Subtheme	Study results describing	Amount of studies	Strength of evidence
Severe and Enduring	Descriptions	Severe	Severe in relation to mental health problems	5	Very strong
		Enduring	Enduring in relation to mental health problems	6	Strong/medium
	Clinical associations with severe and enduring mental health problems	Suicidality	The relation between severe mental health problems and suicidality	7	Strong
		Comorbidity	The relation between SEMHP and comorbidity	4	Medium
Contributing factors	Biological factors	Heredity	The presence of familial (parental) psychiatry in youth with SEMHP	7	Medium
		Age	The biological influence of age on the development or continuation of SEMHP	9	Strong
		Gender	The biological influence of gender (male–female) on the development or continuation of SEMHP	16	Strong/medium
	Psychological factors	Trauma	Psychological stressors and life events, such as maltreatment, abuse, and/or death/loss of a loved one	12	Very strong/strong
	Sociological factors	Socio-economic factors	The role of social stressors in relation to socio-economic status, such as household income, parental education, and parental employment	21	Strong
		Family functioning	The role of social stressors such as family dysfunction and family disruption	14	Strong/medium
		Peer support	The role of social stressors in relationship to/with peers, such as rejection and support	5	Medium
		Ethnic/racial factors	The relation of ethnicity with youth experiencing SEMHP	6	Very strong/strong
Impact	Impact on youth	Academic functioning	Problems in academic functioning (e.g. school dropout) as a result of experiencing SEMHP	8	Very strong/strong
		Psychosocial functioning	Problems in social and emotional functioning (with peers, family) as a result of experiencing SEMHP	11	Very strong/strong
		Hopelessness	Feelings of hopelessness as a result of experiencing SEMHP	5	Medium
		Suicide attempts	Suicide attempts (e.g. out of despair/to feel numb) as a result of experiencing SEMHP	15	Strong
		Substance abuse	Abuse of substances (e.g. drugs and/or alcohol) as a result of experiencing SEMHP	7	Strong/medium
		Criminal behavior	Criminal behavior (e.g. out of despair) as a result of experiencing SEMHP	7	Very strong/strong
	Societal impact	Costs	The effects of youth experiencing SEMHP on society	1	Limited/no evidence
		Policies	The consequences on youth experiencing SEMHP due to societal decisions	2	Medium

### Category 1. Severe and enduring

This category includes subthemes focused on the descriptions of severe and enduring in terms of mental health problems. In addition to the descriptions of severe mental health problems, a separate main theme focusses on clinical associations including suicidality and comorbidity, which were frequently described in relation to severe and enduring.

#### Main theme: Descriptions of severe and enduring mental health problems in youth

##### Descriptions of severe mental health problems

Descriptions of severe in terms of mental health problems [27–31] were related to: (a) ‘a lot’ or ‘extreme’ impairment in daily activities, with serious consequences on the ability to return home, to finish school and to develop personal autonomy to pursue life goals [28–30], in combination with (b) severe/ very severe distress [27, 29, 31]. Moreover, some studies mentioned (c) shortened life expectancy [28]; and (d) symptom recognition by both parents and adolescents [29].

##### Descriptions of enduring mental health problems

Descriptions of enduring in terms of mental health problems included: (a) persistent or recurrent [32, 33]; (b) early onset of mental health problems [27, 32]; (c) duration of illness [29, 34, 35]; (d) duration of treatment: > 6 months [29, 32]. In three studies, mental health problems were reported as enduring after a duration of 12 months [29, 34, 35]. However, in two out of these three studies no association was found between experiencing SEMHP and the duration [34, 35].

#### Main theme: clinical associations with the descriptions of severe and enduring mental health problems

Since suicidality and comorbidity were often described in relation to severe and enduring, we devoted separate themes to these clinical associations. Studies in the subtheme suicidality [29, 30, 34, 36–39] all reported an association between SEMHP and increased suicidality. Studies in the subtheme comorbidity [30, 34, 40, 41] all described the presence of co-occurring psychiatric classifications as part of SEMHP.

### Category 2. Contributing factors

Contributing factors are identified as risk factors for the development or the continuation of SEMHP. These contributing factors were categorized based on the biopsychosocial model [21], including biological factors (e.g. heredity), psychological factors (e.g. trauma), and sociological factors (e.g. socio-economic factors).

#### Main theme: biological factors

##### Heredity

The role of heredity was reported in seven studies [27, 29, 42–46]. In most studies familial psychiatric history was associated with substance abuse problems, major depression, and antisocial personality disorder as the highest risk [27, 29, 43–45]. Although this evidence seems clear, there were two studies [42, 46] that showed no evidence for any association between family history of substance abuse or major depression and youth experiencing SEMHP.

##### Age

The role of age was reported in nine studies [30, 32, 46–52]. However, evidence for the association between age and SEMHP in youth was mixed. First, the influence of age seems to be disorder-specific, for example an increased risk of substance use disorder as youth their age increases [30, 46, 48]. Second, youth were found to be the most vulnerable to co-occurring problems [47], such as suicidal behavior [51]. On the contrary, two studies reported increased mental health problems in younger children [32, 52]. Lastly, some studies reported no differences in age between youth with one specific psychiatric disorder and youth with comorbid psychiatric disorders [46, 49, 50].

##### Gender

The role of gender was reported in 16 studies [27, 30, 38, 39, 41, 43, 45, 46, 48, 51–57]. However, the evidence was mixed, and seemed related to the type of mental health problem (see Table 2). In seven studies, no association was found between gender and mental health problems in youth with SEMHP. More specifically, contradictory results were found for the relation between being female and suicidal behavior/experiencing mixed psychiatric disorders (see Table 2).

**Table 2:** Content analysis-gender

Gender	Type of (mental health) problems and mental disorders	Association	No Association
Female	Suicidal behavior	[27, 51, 55, 57]	[45, 56]
Female	High severity mental health problems	[38]	
Female	Mixed psychiatric disorders	[46, 48, 55]	[28, 39]
Female	Higher prevalence anxiety disorders and eating disorders	[30, 41, 53, 54]	[43]
Female	Higher prevalence emotional problems with a low probability of conduct problems and peer problems, non-occurrence of hyper activity	[52]	
Male	Disruptive disorder		[50]
Male	Increased risk for alcohol and illicit substance use disorders		[30, 39]
Male	Academic problems	[55]	

#### Main theme: psychological factors

##### Trauma

The role of trauma was reported in 12 studies as a contributing factor to SEMHP [27–29, 42–45, 47, 49, 50, 53, 57]. All studies confirmed a substantially elevated exposure to traumatic events for youth with SEMHP. Traumatic exposure for these youth consisted of (a) high rates of a history of abuse and/or neglect (sexual, physical, emotional) [27, 29, 42–45, 49, 57]; (b) more than twice as likely to report (domestic) violence than youth with a single classification or no classification [28, 44, 47, 49, 50]; and (c) death of a loved one [43, 44].

#### Main theme: sociological factors

##### Socio-economic factors

Socio-economic factors were mentioned in 21 studies [27, 28, 30, 32, 36–38, 42, 44, 46, 48, 50, 53, 56–62]. Distinctions between the different types of socioeconomic status in relation to SEMHP can be found in Table 3. Inconsistent results were found for low SES and low household income as a risk factor for the continuation of SEMHP. Furthermore, high SES and high household income were associated as protective factors in youth with SEMHP.

**Table 3:** Content analysis—socio-economic factors

Socio-economic factors in relation to SEMHP	Type of socio-economic factor	Association	No Association
Risk factors	Low SES	[30, 38, 62]	[27, 57]
	Low household income	[28, 30, 36–38, 42, 44, 51, 53, 58, 59, 61]	[32, 46, 57, 60]
	Low parental education	[56, 61]	[57]
	Low parental employment	[50]	[32]
Protective factors	High SES, high household income, high parental education/employment	[32, 38, 48]	

##### Family functioning

The role of family functioning was mentioned in 14 studies [28, 31, 35, 39, 43, 44, 46, 48, 51, 55–57, 60, 61]. Distinctions between the different types of risk factors in family functioning in relation to SEMHP can be found in Table 4. A single-parent home was reported as a risk factor associated with SEMHP, but only for youth with substance use disorder, conduct disorder and major depressive disorder [39, 56].

**Table 4:** Content analysis- family functioning

Family functioning	Type of family functioning	Association	No Association
Risk factors	Separated/divorced parents	[43, 44, 51, 57, 60, 61]	
	Living in a single-parent home	[39, 56]	[57]
	Parents with legal problems	[44]	
	Household members who are very sick	[44]	
	Family experiencing domestic violence	[28, 44]	
	Family conflicts	[39, 46, 48, 55]	
	Lack of family support	[35]	
	Family cohesion	[31]	

##### Peer support

A lack of peer support was mentioned in five studies [31, 39, 45, 53, 63]. Decreased social support in terms of peer-rejection was related to mental health problems in general [31, 39, 45, 63]. Also, one study reported higher quality of social interaction and support of peers in youth with non-specific mental health problems, compared to youth with internalizing and externalizing mental health problems [53].

##### Ethnical factors

Ethnicity was mentioned in six studies [27, 36, 45, 48, 58, 60]. Ethnicity seems to play an role in youth with SEMHP, however the relation remains unclear. This because the evidence was mixed, depending on the type of mental health problem. It was found that anxiety disorders were more prevalent among ethnic minorities [36, 60], while mood disorders were more prevalent among Caucasian youth with parents with higher levels of education [36, 60]. Also, an increased risk of treatment drop-out was found for youth with SEMHP of a foreign nationality [27]. Another study found that Hispanic youth often experience symptoms of a comorbid psychiatric disorder, both internalizing and externalizing [48]. However, two studies found no association between ethnicity and youth experiencing SEMHP [45, 58].

### Category 3. Impact of youth experiencing severe and enduring mental health problems

This category ‘impact’ should be interpreted as the consequences of experiencing SEMHP for youth themselves, their environment and the society they are living in.

#### Main theme: Impact on youth

##### Academic functioning

All eight studies within this theme confirmed problems in academic functioning due to experiencing SEMHP [28–30, 34, 53–55, 60]. These youth experienced academic failure [34], impaired school work [30, 54], problems with school attendance [29, 53] and problems with finishing school [28].

##### Psychosocial functioning

All 11 studies [28–30, 34, 44, 49, 53–55, 57, 60] confirmed problems in psychosocial functioning associated with experiencing SEMHP. Psychosocial functioning included an adolescent’s ability to function socially and emotionally, in which SEMHP were causing e.g. poor quality of life, low self-esteem, and problems with autonomy, family and emotions. Experiencing these psychosocial impairment resulted in a considerable risk potential for an accumulation of complicating factors and future chronicity [30].

##### Hopelessness

Feelings of hopelessness in youth were mentioned in five studies [33, 36–38, 63]. These feelings of hopelessness were higher in youth with SEMHP [33, 36, 38], particularly in youth with severe dysregulated profiles and internalizing problems, in combination with suicidal behavior [33, 36]. Also, hopelessness was associated when youth with SEMHP experienced the following: a negative view of the self, negative view of the world, negative internal attribution, family problems, and/or low positive problem solving orientation [63]. However, there was one study that reported no association with hopelessness after controlling for depression [37].

##### Suicide attempts

Multiple suicide attempts due to experiencing SEMHP was mentioned in 15 studies [27, 29, 34, 36, 37, 39, 44, 45, 47, 50, 51, 54–57]. Suicide attempt as means to regulate intense emotions [37] was associated with SEMHP, especially when anxiety and depression were involved [29, 36, 50, 51, 54–57].

In one study, no differences in attempted suicide rates were found between youth with substance use disorder (SUD) and youth without SUD [57].

##### Substance abuse

Substance abuse in youth was mentioned in seven studies [27, 28, 36, 39, 49, 64, 65]. Five studies found prior mental health problems as a risk factor for comorbid substance use disorder in youth with SEMHP [28, 36, 49, 64, 65], and one study describes substance abuse as means of self-medication [27]. In reverse, one study claimed no unidirectional relation of substance abuse due to experiencing SEMHP, but rather a bi-directional relation, dependent on personal characteristics, the environment and circumstances [39].

##### Criminal behavior

Seven studies confirmed criminal behavior due to experiencing SEMHP [27, 38, 44, 47, 49, 53, 65]. More specifically, 25% of youth with SEMHP reported having been in contact with the legal system [53]. Subgroups most involved were youth with externalizing and overly impulsive problems [44, 53].

#### Main theme: The societal effects of youth with severe and enduring mental health problems

##### Costs

One study mentioned the societal impact of youth with SEMHP in terms of costs [28], namely that the indirect cost of mental health are high due to unemployment/absence from work/chronic sick leaves.

##### Policies

Two studies mentioned the societal impact of youth with SEMHP in terms of policies [28, 53]. These studies underline that treatment for youth with multiple and severe psychiatric disorders became even more complex and less accessible [28, 53]. The studies mentioned two policy issues in developed countries such as Portugal and the Netherlands: (a) there were disparities between political investments in mental health services compared with other areas of healthcare [28]; and (b) limited access to the required services [53].

## Discussion

The aim of this study was to explore the characteristics of youth with severe and enduring mental health problems (SEMHP) in the existing literature. It appears that there is knowledge of contributing factors and the impact of various combinations of classifications on youth functioning in separate publications. However, it seems that no previous study combined these results before in order to describe a target group experiencing heterogenous and severe mental health problems. In this systematic review, we are one of the first to look beyond the classifications and focus on the underlying characteristics of youth with SEMHP.

Our results indicate that youth with SEMHP are characterized by co-occurring mental health problems, frequently in combination with pervasive suicidality. The severity of their mental health problems are interpreted by the experienced serious impairment in functioning in combination with severe distress. The endurance of their mental health problems is interpreted by the recurrent character often with an early onset. An important contributing factor associated with SEMHP was an underlying trauma, which seems to be a pervasive factor. Also, a low household income; problems in family functioning, such as separated parents and family conflict; and lack of support by family and peers were identified as contributing factors to SEMHP. As a result, youths’ development is hindered on multiple domains such as academic and psychosocial functioning with often reported substance abuse, criminal behavior and deep feelings of hopelessness.

Overall, several studies reviewed outline a pervasive pattern of dysfunction in multiple domains leading to a detrimental impact on youths’ daily life. Even more, classifications do not seem to describe the core of SEMHP. By solely focusing on the classifications, without attention for the underlying mental health problems, youth may feel unheard and unrecognized. The section below discusses the most relevant characteristics of youth with SEMHP per category in light of this review results, followed by a discussion of future directions and strengths and limitations.

### Severe and enduring

In the current mental health system, the concept of severity in terms of mental health problems often refers to the intensity of symptoms using a ranking system [9, 66, 67]. However, in this study we suggest a different interpretation of severity, focusing on the level of impairment and distress experienced by youth with SEMHP. Similar to the results of Fonagy et al. [9], we identified clinical associations with SEMHP including a varying range of co-occurring mental health problems, often in combination with suicidality, but also with deep feelings of hopelessness. This implies that no specific DSM classification can be ascribed to the target group of youth with SEMHP, and that there is a need for a different description. Moreover, to gain a better understanding of youth with SEMHP, more research is needed into co-occurring mental health problems. In addition to the P-factor theory, currently different approaches [4, 68], such as Hierarchical Taxonomy of Psychopathology (HiTOP) model [69] and the Research Domain Criteria (RDoc) system [68], focus on the underlying connections between conditions in a dimensional model [69], while similarly taking into account explanatory underlying transdiagnostic mechanisms [4, 68]. Future studies should explore to what extent these approaches fit the target group whose characteristics we have identified.

### Contributing factors

Our results show a pervasive pattern of (childhood) trauma associated with youth with SEMHP. This finding is supported by various prior studies through the years, in combination with the effects of parental mental illness on youth [9, 70, 71]. Therefore, in order to provide adequate mental healthcare for youth with SEMHP, identifying and treating trauma in both youth and their parents is crucial. This requires sufficient time, skilled practitioners and resources. Moreover, attention should be paid to psychosocial environment (e.g. lack of support by family and peers) of youth with SEMHP. While for most youth puberty is a time of detachment from parents and greater reliance on peers [72], for youth without strong social connections, puberty is a high risk period which can be the beginning of severe and enduring mental health problems [9, 73, 74]. Although the underlying trauma and social connections seems crucial in youth with SEMHP, it is lacking in the current description of the SEMHP population [10]. In diagnosing youth with SEMHP, a holistic approach is needed including youths’ psychosocial support system so that factors such as trauma, peer rejection and/or family conflicts are identified faster and youth can be supported better.

### Impact

Experiencing SEMHP has an enormous impact on youths’ feelings and behavior. Similar to the description of Delespaul [10] our results show severe disabilities in social functioning for youth with SEMHP, such as hindered academic and psychosocial functioning combined with poor quality of life, low self-esteem, suicidal behavior and deep feelings of hopelessness. The impact of SEMHP on youth seem to perpetuate the problem, where the disability is both cause and effect [10]. This vicious cycle is a considerable risk potential for an accumulation of complicating factors and future chronicity [30]. Therefore, practitioners should not only focus on the symptoms related to illness, but also (and maybe preferably) on the interaction of symptoms with functioning in different areas of life. This interaction may also differ between individuals, and that is why it is so important to start a conversation with youth themselves, instead of (only) targeted treatment based on a protocol.

### Strengths and limitations

This review has several strengths. First, we reduced the risk of reporting bias by prospectively registering our review protocol in PROSPERO. Second, to increase applicability and generalizability a wide range of (a) mental health problems; (b) perspectives; (c) mental health care settings were included. Third, we reduced the selection bias by independent screening of the articles by two researchers. Fourth, in order to guarantee the quality, we critically appraised the individual studies and assessed the strength of evidence per subtheme. Only two articles that were included were of low quality and for only one subtheme the evidence appeared weak due to lack of studies.

Undeniably, our results should be interpreted in the context of various limitations. Our search terms were very broad without clear demarcation for the terms severe and enduring, making it difficult to measure whether studies were about the same group. We decided to refer to these youth as youth with severe and enduring mental health problems (SEMHP). In doing so, we did not apply any cut-off scores for severity in terms of grading scores, such as the Global Assessment of Functioning (GAF) score [75]. We aimed to go beyond the traditional way of classifying symptoms based on a list of criteria and time restraints, as the DSM-5 does [4]. We also decided to explore the term enduring without any cut-off score for the duration of mental health problems. Hence, the lack of numeric scores to assess which study should be included, can be seen as a limitation of this review. However, we believe that our carefully chosen set of descriptive inclusion criteria fits the heterogeneous nature of the SEMHP population. Furthermore, our target group experiences heterogenous mental health problems resulting into inclusion of studies with various mental health problems with unquestionably different outcomes and expressions. However, because we have not limited ourselves to a specific mental health problem or one combination of comorbid mental health problems, we can learn more about any common denominator, and that is what makes this study so unique. Moreover, while the screening process and the thematic analysis were performed with multiple researchers, the coding process has been done by only one researcher. Despite supervision by a senior researcher, this is a limitation of this paper because it might add subjectivity to the results*.* In addition, we have made a distinction between factors that affect the development and continuation of SEMHP (contributing factors) and the consequences of experiencing SEMHP (impact). We have tended to describe directional relations, whereas there is no evidence for this. This review shows that there is no specific evidence for a causal relationship, however we do know that there is an interaction between these factors, consequences and SEMHP. That is the strength of this study, as well as the complexity. Moreover, with respect to ethnicity, our results should be interpreted with caution. None of our articles reported third-world countries which undoubtably also have youth with severe and enduring mental health problems [76]. However, for Western youth, interpretation of the data seems sufficient. Finally, since this review is not a meta-analysis, we were unable to draw conclusions about causal relationships, strength of the associations, or whether one factor is more important than another. Therefore, further research of the personal and environmental factors is needed to identify potential moderators.

## Conclusion

This review is the first to thematically explore and describe characteristics of youth with severe and enduring mental health problems (SEMHP). While the traditional classification system has long been used to describe mental problems, this review suggests shifting the focus to a more descriptive diagnoses including personal and environmental factors. In particular, trauma and suicidality seem key elements in understanding youth with SEMHP and therefore should be included in diagnostic decision making. Also, the pervasive patterns of dysfunction in multiple domains leading to a crucial impact, such as hindered academic and psychosocial functioning, substance abuse and deep feelings of hopelessness should be taken into account by practice. In order to understand the vicious cycle of (mental health) problems experienced by youth with SEMHP, more research is needed into the comorbid mental health problems and what underlies them. This should be done in cooperation with these youth.

### Supplementary Information

Below is the link to the electronic supplementary material.

Supplementary file1 (DOCX 19 KB)

Supplementary file2 (DOCX 81 KB)

Supplementary file3 (DOCX 27 KB)

## Data Availability

The datasets generated and analysed during the current study are available from the corresponding author on reasonable request.
