# Supplementary material for: A systematic review exploring characteristics of youth with severe and enduring mental health problems (SEMHP)
Source: Eur Child Adolesc Psychiatry. 2023 Apr 24;33(5):1313–25. doi: 10.1007/s00787-023-02216-6 (PMC11098915; doi:10.1007/s00787-023-02216-6)
Supplement: Supplementary file 1 — Supplementary file1 (DOCX 19 KB) [file 787_2023_2216_MOESM1_ESM.docx]

**Appendix A. Search strategy**

**PubMed**

*Pubmed search strategy on 18 January 2023: 268*

(((("child"[majr] OR "child"[ti] OR "children"[ti] OR "young adult"[majr] OR "adolescent"[majr] OR "schoolchild"[ti] OR "schoolchildren"[ti] OR "youngster"[ti] OR "youngsters"[ti] OR "boy"[ti] OR "boys"[ti] OR "girl"[ti] OR "girls"[ti] OR "Adolescent"[majr] OR "adolescent"[ti] OR "adolescents"[ti] OR "adolescence"[ti] OR "schoolage"[ti] OR "schoolboy"[ti] OR "schoolboys"[ti] OR "schoolgirl"[ti] OR "schoolgirls"[ti] OR "prepuber"[ti] OR "prepubers"[ti] OR "prepuberty"[ti] OR "puber"[ti] OR "pubers"[ti] OR "puberty"[ti] OR "teenager"[ti] OR "teenagers"[ti] OR "teens"[ti] OR "youth"[ti] OR "youths"[ti] OR "underaged"[ti] OR "under-aged"[ti] OR "Pediatrics"[majr] OR "Pediatric"[ti] OR "Pediatrics"[ti] OR "Paediatric"[ti] OR "Paediatrics"[ti])) AND (("Mental Disorders"[majr:noexp] OR "Mental Disorder*"[ti] OR "mental health problem*"[ti] OR "psychiatric disorder*"[ti] OR "mental health difficult*"[ti] OR "mental disease*"[ti] OR "mental illness*"[ti] OR "psychiatric disease*"[ti] OR "psychiatric illness*"[ti] OR "behavior disorder*"[ti] OR "behaviour disorder*"[ti] OR "behavioral disorder*"[ti] OR "behavioural disorder*"[ti] OR "behavior disorder*"[ti] OR "behaviour disorder*"[ti] OR "behavioral disorder*"[ti] OR "behavioural disorder*"[ti] OR "behavior problem*"[ti] OR "behaviour problem*"[ti] OR "behavioral problem*"[ti] OR "behavioural problem*"[ti])) OR "internalizing disoder*"[ti] OR " externalizing disoder*"[ti] OR "thought disorder*"[ti] AND (("severe"[tiab] OR "suffering"[tiab] OR "hardship*"[tiab] OR "persisten*"[tiab] OR "persisting"[tiab] OR "enduring"[tiab] OR "endures"[tiab] OR "endure"[tiab] OR "endured"[tiab] OR "early onset"[tiab] OR "lifetime"[tiab] OR " life time"[tiab] OR "life-long"[tiab] OR "lifelong"[tiab]) AND ("predict*"[tiab] OR "risk"[majr] OR "riskfactor*"[tiab] OR "preventi*"[tiab] OR "risk"[tiab] OR "risks"[tiab] OR "Child Abuse"[Majr] OR "Child Abuse"[tiab] OR "maltreatment*"[tiab] OR "maltreated"[tiab] OR "Bullying"[Majr] OR "bully*"[tiab] OR "bullie*"[tiab] OR "Self-concept"[Majr] OR "self-esteem*"[tiab] OR "trauma"[tiab] OR "traumas"[tiab] OR "psychotrauma*"[tiab] OR "attach*"[tiab] OR "Social Support"[Majr] OR "social support*"[tiab] OR "belongingness"[tiab] OR "epistemic trust*"[tiab] OR "social bind*"[tiab] OR "hyper vigil*"[tiab] OR "hypervigil*"[tiab] OR "suicide"[majr] OR "suicid*"[tiab] OR "self harm"[majr] OR "treatment refusal"[majr] OR "biopsychosocial"[tiab] OR "psychosocial"[tiab] OR (("therap*"[ti] OR "treatment*"[ti]) AND ("resist*"[ti] OR "refus*"[ti])) OR ("suffer*"[ti] AND "intense"[ti]) OR "parental abus*"[tw] OR ("parent*"[ti] AND "abus*"[ti] AND ("acohol*"[ti] OR "drug"[ti] OR "drugs"[ti] OR "substance*"[ti])) OR ("parent*"[ti] AND ("behavior*"[ti] OR "behaviour*"[ti] OR "stress"[ti] OR "low income*"[ti])) OR "family history"[tiab] OR "adoption"[majr] OR "adopt*"[tiab]) OR "demograph*"[tiab] OR "socioeconomic*"[tiab] OR "socio-economic*"[tiab] OR ("low"[tiab] AND "educat*"[tiab]) AND ("complex"[ti] OR "comorbidity"[majr] OR "comorbid*"[ti] OR "multimorbid*"[ti] OR "multi morbid*"[ti] OR "multi-morbid*"[ti] OR ("multiple"[ti] AND ("disorder*"[ti] OR "illness*"[ti] OR "morbidit*"[ti]))))) OR ((("child"[majr] OR "child"[ti] OR "children"[ti] OR "young adult"[majr] OR "adolescent"[majr] OR "schoolchild"[ti] OR "schoolchildren"[ti] OR "youngster"[ti] OR "youngsters"[ti] OR "boy"[ti] OR "boys"[ti] OR "girl"[ti] OR "girls"[ti] OR "Adolescent"[majr] OR "adolescent"[ti] OR "adolescents"[ti] OR "adolescence"[ti] OR "schoolage"[ti] OR "schoolboy"[ti] OR "schoolboys"[ti] OR "schoolgirl"[ti] OR "schoolgirls"[ti] OR "prepuber"[ti] OR "prepubers"[ti] OR "prepuberty"[ti] OR "puber"[ti] OR "pubers"[ti] OR "puberty"[ti] OR "teenager"[ti] OR "teenagers"[ti] OR "teens"[ti] OR "youth"[ti] OR "youths"[ti] OR "underaged"[ti] OR "under-aged"[ti] OR "Pediatrics"[majr] OR "Pediatric"[ti] OR "Pediatrics"[ti] OR "Paediatric"[ti] OR "Paediatrics"[ti])) AND (("Mental Disorders"[majr:noexp] OR "Mental Disorder*"[ti] OR "mental health problem*"[ti] OR "psychiatric disorder*"[ti] OR "mental health difficult*"[ti] OR "mental disease*"[ti] OR "mental illness*"[ti] OR "psychiatric disease*"[ti] OR "psychiatric illness*"[ti] OR "behavior disorder*"[ti] OR "behaviour disorder*"[ti] OR "behavioral disorder*"[ti] OR "behavioural disorder*"[ti] OR "behavior disorder*"[ti] OR "behaviour disorder*"[ti] OR "behavioral disorder*"[ti] OR "behavioural disorder*"[ti] OR "behavior problem*"[ti] OR "behaviour problem*"[ti] OR "behavioral problem*"[ti] OR "behavioural problem*"[ti])) AND (("severe"[tiab] OR "suffering"[tiab] OR "hardship*"[tiab] OR "persisten*"[tiab] OR "persisting"[tiab] OR "enduring"[tiab] OR "endures"[tiab] OR "endure"[tiab] OR "endured"[tiab] OR "early onset"[tiab] OR "lifetime"[tiab] OR " life time"[tiab] OR "life long"[tiab] OR "lifelong"[tiab]) AND ("predict*"[ti] OR "risk"[majr] OR "riskfactor*"[ti] OR "preventi*"[ti] OR "risk"[ti] OR "risks"[ti] OR "Child Abuse"[Majr] OR "Child Abuse"[ti] OR "maltreatment*"[ti] OR "maltreated"[ti] OR "Bullying"[Majr] OR "bully*"[ti] OR "bullie*"[ti] OR "Self Concept"[Majr] OR "self-esteem*"[ti] OR "trauma"[ti] OR "traumas"[ti] OR "psychotrauma*"[ti] OR "attach*"[ti] OR "Social Support"[Majr] OR "social support*"[ti] OR "belongingness"[ti] OR "epistemic trust*"[ti] OR "social bind*"[ti] OR "hyper vigil*"[ti] OR "hypervigil*"[ti] OR "suicide"[majr] OR "suicid*"[ti] OR "treatment refusal"[majr] OR "biopsychosocial"[tiab] OR "psychosocial"[tiab] OR (("therap*"[ti] OR "treatment*"[ti]) AND ("resist*"[ti] OR "refus*"[ti])) OR ("suffer*"[ti] AND "intense"[ti]) OR "parental abus*"[tw] OR ("parent*"[ti] AND "abus*"[ti] AND ("acohol*"[ti] OR "drug"[ti] OR "drugs"[ti] OR "substance*"[ti])) OR ("parent*"[ti] AND ("behavior*"[ti] OR "behaviour*"[ti] OR "stress"[ti] OR "low income*"[ti])) OR "family history"[ti] OR "adoption"[majr] OR "adopt*"[ti]) OR "demograph*"[ti] OR "socioeconomic*"[ti] OR "socio-economic*"[ti] OR ("low"[ti] AND "educat*"[ti]) AND ("complex"[tiab] OR "comorbidity"[majr] OR "comorbid*"[tiab] OR "multimorbid*"[tiab] OR "multi morbid*"[tiab] OR "multi-morbid*"[tiab] OR ("multiple"[tiab] AND ("disorder*"[tiab] OR "illness*"[tiab] OR "morbidit*"[tiab])))))) OR ((("child"[majr] OR "child"[ti] OR "children"[ti] OR "young adult"[majr] OR "adolescent"[majr] OR "schoolchild"[ti] OR "schoolchildren"[ti] OR "youngster"[ti] OR "youngsters"[ti] OR "boy"[ti] OR "boys"[ti] OR "girl"[ti] OR "girls"[ti] OR "Adolescent"[majr] OR "adolescent"[ti] OR "adolescents"[ti] OR "adolescence"[ti] OR "schoolage"[ti] OR "schoolboy"[ti] OR "schoolboys"[ti] OR "schoolgirl"[ti] OR "schoolgirls"[ti] OR "prepuber"[ti] OR "prepubers"[ti] OR "prepuberty"[ti] OR "puber"[ti] OR "pubers"[ti] OR "puberty"[ti] OR "teenager"[ti] OR "teenagers"[ti] OR "teens"[ti] OR "youth"[ti] OR "youths"[ti] OR "underaged"[ti] OR "under-aged"[ti] OR "Pediatrics"[majr] OR "Pediatric"[ti] OR "Pediatrics"[ti] OR "Paediatric"[ti] OR "Paediatrics"[ti])) AND (("Mental Disorders"[majr] OR "Mental Disorder*"[ti] OR "mental health problem*"[ti] OR "psychiatric disorder*"[ti] OR "mental health difficult*"[ti] OR "mental disease*"[ti] OR "mental illness*"[ti] OR "psychiatric disease*"[ti] OR "psychiatric illness*"[ti] OR "behavior disorder*"[ti] OR "behaviour disorder*"[ti] OR "behavioral disorder*"[ti] OR "behavioural disorder*"[ti] OR "behavior disorder*"[ti] OR "behaviour disorder*"[ti] OR "behavioral disorder*"[ti] OR "behavioural disorder*"[ti] OR "behavior problem*"[ti] OR "behaviour problem*"[ti] OR "behavioral problem*"[ti] OR "behavioural problem*"[ti] OR "anxiety disorder*"[ti] OR "PTSD"[ti] OR "posttraumatic stress disorder*"[ti] OR "post traumatic stress disorder*"[ti] OR "post-traumatic stress disorder*"[ti] OR "eating disorder*"[ti] OR "bipolar disorder*"[ti] OR "impulse control"[ti] OR "conductive disorder*"[ti] OR "disruptive disorder*"[ti] OR "oppositional defiant disorder*"[ti] OR ("autis*"[ti] AND "disorder*"[ti]) OR "ADHD"[ti] OR "Attention Deficit Disorder*"[ti] OR "mood disorder*"[ti] OR "depression*"[ti] OR "depressive disorder*"[ti] OR "schizophreni*"[ti] OR "psychos*"[ti] OR "psychotic"[ti] OR "personality disorder*"[ti] OR "substance related disorder*"[ti] OR "substance-related disorder*"[ti] OR "substance disorder*"[ti] OR "alcohol disorder*"[ti] OR "alcohol abus*"[ti] OR ("Marijuana"[ti] AND "abus*"[ti]) OR "Amphetamine-Related Disorder*"[ti] OR "Amphetamine Related Disorder*"[ti] OR ("XTC"[ti] AND "abus*"[ti]) OR "drug abus*"[ti] OR "drug use disorder*"[ti])) AND (("sever*"[ti] OR "intense"[ti] OR "suffering"[ti] OR "hardship*"[ti] OR "persisten*"[ti] OR "persisting"[ti] OR "enduring"[ti] OR "endures"[ti] OR "endure"[ti] OR "endured"[ti] OR "early onset"[ti] OR "lifetime"[ti] OR " life time"[ti] OR "life long"[ti] OR "lifelong"[ti]) AND ("predict*"[ti] OR "risk"[majr] OR "riskfactor*"[ti] OR "preventi*"[ti] OR "risk"[ti] OR "risks"[ti] OR "Child Abuse"[Majr] OR "Child Abuse"[ti] OR "maltreatment*"[ti] OR "maltreated"[ti] OR "Bullying"[Majr] OR "bully*"[ti] OR "bullie*"[ti] OR "Self Concept"[Majr] OR "self-esteem*"[ti] OR "trauma"[ti] OR "traumas"[ti] OR "psychotrauma*"[ti] OR "attach*"[ti] OR "Social Support"[Majr] OR "social support*"[ti] OR "belongingness"[ti] OR "epistemic trust*"[ti] OR "social bind*"[ti] OR "hyper vigil*"[ti] OR "hypervigil*"[ti] OR "suicide"[majr] OR "suicid*"[ti] OR "treatment refusal"[majr] OR "biopsychosocial"[tiab] OR "psychosocial"[tiab] OR (("therapy"[ti] OR "treatment*"[ti] OR "therapeutics"[ti]) AND ("resist*"[ti] OR "refus*"[ti])) OR ("suffer*"[ti] AND "intense"[ti]) OR ("parent*"[ti] AND "abus*"[ti] AND ("acohol*"[ti] OR "drug"[ti] OR "drugs"[ti] OR "substance*"[ti])) OR ("parent*"[ti] AND ("behavior*"[ti] OR "behaviour*"[ti] OR "stress"[ti] OR "low income*"[ti])) OR "family history"[ti] OR "adoption"[majr] OR "adopt*"[ti]) OR "demograph*"[ti] OR "socioeconomic*"[ti] OR "socio-economic*"[ti] OR ("low"[ti] AND "educat*"[ti]) AND ("complex"[ti] OR "comorbidity"[majr] OR "comorbid*"[ti] OR "multimorbid*"[ti] OR "multi morbid*"[ti] OR "multi-morbid*"[ti] OR ("multiple"[ti] AND ("disorder*"[ti] OR "illness*"[ti] OR "morbidit*"[ti])))))

**Web of Sciences**
*Web of Sciences strategy on 18 January 2023: 212*

(TI=(((("child" OR "child" OR "children" OR "young adult" OR "adolescent" OR "schoolchild" OR "schoolchildren" OR "youngster" OR "youngsters" OR "boy" OR "boys" OR "girl" OR "girls" OR "Adolescent" OR "adolescent" OR "adolescents" OR "adolescence" OR "schoolage" OR "schoolboy" OR "schoolboys" OR "schoolgirl" OR "schoolgirls" OR "prepuber" OR "prepubers" OR "prepuberty" OR "puber" OR "pubers" OR "puberty" OR "teenager" OR "teenagers" OR "teens" OR "youth" OR "youths" OR "underaged" OR "under-aged" OR "Pediatrics" OR "Pediatric" OR "Pediatrics" OR "Paediatric" OR "Paediatrics")) AND (("Mental Disorders" OR "Mental Disorder*" OR "mental health problem*" OR "psychiatric disorder*" OR "mental health difficult*" OR "mental disease*" OR "mental illness*" OR "psychiatric disease*" OR "psychiatric illness*" OR "behavior disorder*" OR "behaviour disorder*" OR "behavioral disorder*" OR "behavioural disorder*" OR "behavior disorder*" OR "behaviour disorder*" OR "behavioral disorder*" OR "behavioural disorder*" OR "behavior problem*" OR "behaviour problem*" OR "behavioral problem*" OR "behavioural problem*" OR "internalizing disorder*" OR "externalizing disorder*" OR "thought disorder*" OR "anxiety disorder*" OR "eating disorder*" OR "PTSD" OR "posttraumatic stress disorder*" OR "post traumatic stress disorder*" OR "post-traumatic stress disorder*" OR "bipolar disorder*" OR "impulse control" OR "conductive disorder*" OR "disruptive disorder*" OR "oppositional defiant disorder*" OR ("autis*" AND "disorder*") OR "ADHD" OR "Attention Deficit Disorder*" OR "mood disorder*" OR "depression*" OR "depressive disorder*" OR "schizophreni*" OR "psychos*" OR "psychotic" OR "personality disorder*" OR "substance related disorder*" OR "substance-related disorder*" OR "substance disorder*" OR "alcohol disorder*" OR "alcohol abus*" OR ("Marijuana" AND "abus*") OR "Amphetamine-Related Disorder*" OR "Amphetamine Related Disorder*" OR ("XTC" AND "abus*") OR "drug abus*" OR "drug use disorder*"))) AND (("sever*" OR "intense" OR "suffering" OR "hardship*" OR "persisten*" OR "persisting" OR "enduring" OR "endures" OR "endure" OR "endured" OR "early onset" OR "lifetime" OR "life time" OR "life long" OR "lifelong") AND ("predict*" OR "risk" OR "riskfactor*" OR "preventi*" OR "risk" OR "risks" OR "Child Abuse" OR "Child Abuse" OR "maltreatment*" OR "maltreated" OR "Bullying" OR "bully*" OR "bullie*" OR "Self Concept" OR "self-esteem*" OR "trauma" OR "traumas" OR "psychotrauma*" OR "attach*" OR "Social Support" OR "social support*" OR "belongingness" OR "epistemic trust*" OR "social bind*" OR "hyper vigil*" OR "hypervigil*" OR "suicide" OR "suicid*" OR "treatment refusal" OR "biopsychosocial" OR "psychosocial" OR (("therapy" OR "treatment*" OR "therapeutics") AND ("resist*" OR "refus*")) OR ("suffer*" AND "intense") OR ("parent*" AND "abus*" AND ("acohol*" OR "drug" OR "drugs" OR "substance*")) OR ("parent*" AND ("behavior*" OR "behaviour*" OR "stress" OR "low income*")) OR "family history" OR "adopt*" OR "demograph*" OR "socioeconomic*" OR "socio-economic*" OR ("low" AND "educat*")) AND ("complex" OR "comorbidity" OR "comorbid*" OR "multimorbid*" OR "multi morbid*" OR "multi-morbid*" OR ("multiple" AND ("disorder*" OR "illness*" OR "morbidit*")))))) OR (TI=("child" OR "child" OR "children" OR "young adult" OR "adolescent" OR "schoolchild" OR "schoolchildren" OR "youngster" OR "youngsters" OR "boy" OR "boys" OR "girl" OR "girls" OR "Adolescent" OR "adolescent" OR "adolescents" OR "adolescence" OR "schoolage" OR "schoolboy" OR "schoolboys" OR "schoolgirl" OR "schoolgirls" OR "prepuber" OR "prepubers" OR "prepuberty" OR "puber" OR "pubers" OR "puberty" OR "teenager" OR "teenagers" OR "teens" OR "youth" OR "youths" OR "underaged" OR "under-aged" OR "Pediatrics" OR "Pediatric" OR "Pediatrics" OR "Paediatric" OR "Paediatrics") AND TI=("Mental Disorders" OR "Mental Disorder*" OR "mental health problem*" OR "psychiatric disorder*" OR "mental health difficult*" OR "mental disease*" OR "mental illness*" OR "psychiatric disease*" OR "psychiatric illness*" OR "behavior disorder*" OR "behaviour disorder*" OR "behavioral disorder*" OR "behavioural disorder*" OR "behavior disorder*" OR "behaviour disorder*" OR "behavioral disorder*" OR "behavioural disorder*" OR "behavior problem*" OR "behaviour problem*" OR "behavioral problem*" OR "behavioural problem*") AND TS=("severe" OR "suffering" OR "hardship*" OR "persisten*" OR "persisting" OR "enduring" OR "endures" OR "endure" OR "endured" OR "early onset" OR "lifetime" OR " life time" OR "life long" OR "lifelong") AND (TS="predict*" OR TS="riskfactor*" OR TS="preventi*" OR TS="risk" OR TS="risks" OR TS="Child Abuse" OR TS="maltreatment*" OR TS="maltreated" OR TS="bully*" OR TS="bullie*" OR TS="Self Concept" OR TS="self-esteem*" OR TS="trauma" OR TS="traumas" OR TS="psychotrauma*" OR TS="attach*" OR TS="social support*" OR TS="belongingness" OR TS="epistemic trust*" OR TS="social bind*" OR TS="hyper vigil*" OR TS="hypervigil*" OR TS="suicid*" OR TS="treatment refusal" OR TS="biopsychosocial" OR TS="psychosocial" OR TI=(("therap*" OR "treatment*") AND ("resist*" OR "refus*")) OR TI=("suffer*" AND "intense") OR TS="parental abus*" OR TI=("parent*" AND "abus*" AND ("acohol*" OR "drug" OR "drugs" OR "substance*")) OR TI=("parent*" AND ("behavior*" OR "behaviour*" OR "stress" OR "low income*")) OR TS="family history" OR TS="adopt*" OR TS="demograph*" OR TS="socioeconomic*" OR TS="socio-economic*" OR TI=("low" AND "educat*")) AND (TS="complex" OR TS="comorbidity" OR TS="comorbid*" OR TS="multimorbid*" OR TS="multi morbid*" OR TS="multi-morbid*" OR (TI="multiple" AND (TI="disorder*" OR TI="illness*" OR TI="morbidit*"))))

**PSYCHINFO**

*PsychInfo search strategy on 18 January 2023: 193*

(TI(((("child" OR "child" OR "children" OR "young adult" OR "adolescent" OR "schoolchild" OR "schoolchildren" OR "youngster" OR "youngsters" OR "boy" OR "boys" OR "girl" OR "girls" OR "Adolescent" OR "adolescent" OR "adolescents" OR "adolescence" OR "schoolage" OR "schoolboy" OR "schoolboys" OR "schoolgirl" OR "schoolgirls" OR "prepuber" OR "prepubers" OR "prepuberty" OR "puber" OR "pubers" OR "puberty" OR "teenager" OR "teenagers" OR "teens" OR "youth" OR "youths" OR "underaged" OR "under-aged" OR "Pediatrics" OR "Pediatric" OR "Pediatrics" OR "Paediatric" OR "Paediatrics")) AND (("Mental Disorders" OR "Mental Disorder*" OR "mental health problem*" OR "psychiatric disorder*" OR "mental health difficult*" OR "mental disease*" OR "mental illness*" OR "psychiatric disease*" OR "psychiatric illness*" OR "behavior disorder*" OR "behaviour disorder*" OR "behavioral disorder*" OR "behavioural disorder*" OR "behavior disorder*" OR "behaviour disorder*" OR "behavioral disorder*" OR "behavioural disorder*" OR "behavior problem*" OR "behaviour problem*" OR "behavioral problem*" OR "behavioural problem*" OR "internalizing disorder*" OR "externalizing disorder*" OR "thought disorder*" OR "anxiety disorder*" OR "eating disorder*" OR "PTSD" OR "posttraumatic stress disorder*" OR "post traumatic stress disorder*" OR "post-traumatic stress disorder*" OR "bipolar disorder*" OR "impulse control" OR "conductive disorder*" OR "disruptive disorder*" OR "oppositional defiant disorder*" OR ("autis*" AND "disorder*") OR "ADHD" OR "Attention Deficit Disorder*" OR "mood disorder*" OR "depression*" OR "depressive disorder*" OR "schizophreni*" OR "psychos*" OR "psychotic" OR "personality disorder*" OR "substance related disorder*" OR "substance-related disorder*" OR "substance disorder*" OR "alcohol disorder*" OR "alcohol abus*" OR ("Marijuana" AND "abus*") OR "Amphetamine-Related Disorder*" OR "Amphetamine Related Disorder*" OR ("XTC" AND "abus*") OR "drug abus*" OR "drug use disorder*"))) AND (("sever*" OR "intense" OR "suffering" OR "hardship*" OR "persisten*" OR "persisting" OR "enduring" OR "endures" OR "endure" OR "endured" OR "early onset" OR "lifetime" OR "life time" OR "life long" OR "lifelong") AND ("predict*" OR "risk" OR "riskfactor*" OR "preventi*" OR "risk" OR "risks" OR "Child Abuse" OR "Child Abuse" OR "maltreatment*" OR "maltreated" OR "Bullying" OR "bully*" OR "bullie*" OR "Self Concept" OR "self-esteem*" OR "trauma" OR "traumas" OR "psychotrauma*" OR "attach*" OR "Social Support" OR "social support*" OR "belongingness" OR "epistemic trust*" OR "social bind*" OR "hyper vigil*" OR "hypervigil*" OR "suicide" OR "suicid*" OR "treatment refusal" OR "biopsychosocial" OR "psychosocial" OR (("therapy" OR "treatment*" OR "therapeutics") AND ("resist*" OR "refus*")) OR ("suffer*" AND "intense") OR ("parent*" AND "abus*" AND ("acohol*" OR "drug" OR "drugs" OR "substance*")) OR ("parent*" AND ("behavior*" OR "behaviour*" OR "stress" OR "low income*")) OR "family history" OR "adopt*" OR "demograph*" OR "socioeconomic*" OR "socio-economic*" OR ("low" AND "educat*")) AND ("complex" OR "comorbidity" OR "comorbid*" OR "multimorbid*" OR "multi morbid*" OR "multi-morbid*" OR ("multiple" AND ("disorder*" OR "illness*" OR "morbidit*")))))) OR (TI("child" OR "child" OR "children" OR "young adult" OR "adolescent" OR "schoolchild" OR "schoolchildren" OR "youngster" OR "youngsters" OR "boy" OR "boys" OR "girl" OR "girls" OR "Adolescent" OR "adolescent" OR "adolescents" OR "adolescence" OR "schoolage" OR "schoolboy" OR "schoolboys" OR "schoolgirl" OR "schoolgirls" OR "prepuber" OR "prepubers" OR "prepuberty" OR "puber" OR "pubers" OR "puberty" OR "teenager" OR "teenagers" OR "teens" OR "youth" OR "youths" OR "underaged" OR "under-aged" OR "Pediatrics" OR "Pediatric" OR "Pediatrics" OR "Paediatric" OR "Paediatrics") AND TI("Mental Disorders" OR "Mental Disorder*" OR "mental health problem*" OR "psychiatric disorder*" OR "mental health difficult*" OR "mental disease*" OR "mental illness*" OR "psychiatric disease*" OR "psychiatric illness*" OR "behavior disorder*" OR "behaviour disorder*" OR "behavioral disorder*" OR "behavioural disorder*" OR "behavior disorder*" OR "behaviour disorder*" OR "behavioral disorder*" OR "behavioural disorder*" OR "behavior problem*" OR "behaviour problem*" OR "behavioral problem*" OR "behavioural problem*") AND TX("severe" OR "suffering" OR "hardship*" OR "persisten*" OR "persisting" OR "enduring" OR "endures" OR "endure" OR "endured" OR "early onset" OR "lifetime" OR " life time" OR "life long" OR "lifelong") AND (TX"predict*" OR TX"riskfactor*" OR TX"preventi*" OR TX"risk" OR TX"risks" OR TX"Child Abuse" OR TX"maltreatment*" OR TX"maltreated" OR TX"bully*" OR TX"bullie*" OR TX"Self Concept" OR TX"self-esteem*" OR TX"trauma" OR TX"traumas" OR TX"psychotrauma*" OR TX"attach*" OR TX"social support*" OR TX"belongingness" OR TX"epistemic trust*" OR TX"social bind*" OR TX"hyper vigil*" OR TX"hypervigil*" OR TX"suicid*" OR TX"treatment refusal" OR TX"biopsychosocial" OR TX"psychosocial" OR TI(("therap*" OR "treatment*") AND ("resist*" OR "refus*")) OR TI("suffer*" AND "intense") OR TX"parental abus*" OR TI("parent*" AND "abus*" AND ("alcohol*" OR "drug" OR "drugs" OR "substance*")) OR TI("parent*" AND ("behavior*" OR "behaviour*" OR "stress" OR "low income*")) OR TX"family history" OR TX"adopt*" OR TX"demograph*" OR TX"socioeconomic*" OR TX"socio-economic*" OR TI("low" AND "educat*")) AND (TX"complex" OR TX"comorbidity" OR TX"comorbid*" OR TX"multimorbid*" OR TX"multi morbid*" OR TX"multi-morbid*" OR (TI"multiple" AND (TI"disorder*" OR TI"illness*" OR TI"morbidit*"))))

**COCHRANE**

*Cochrane search strategy on 18 Janaury 2023: 1605*

((((("child" OR "child" OR "children" OR "young adult" OR "adolescent" OR "schoolchild" OR "schoolchildren" OR "youngster" OR "youngsters" OR "boy" OR "boys" OR "girl" OR "girls" OR "Adolescent" OR "adolescent" OR "adolescents" OR "adolescence" OR "schoolage" OR "schoolboy" OR "schoolboys" OR "schoolgirl" OR "schoolgirls" OR "prepuber" OR "prepubers" OR "prepuberty" OR "puber" OR "pubers" OR "puberty" OR "teenager" OR "teenagers" OR "teens" OR "youth" OR "youths" OR "underaged" OR "under-aged" OR "Pediatrics" OR "Pediatric" OR "Pediatrics" OR "Paediatric" OR "Paediatrics")) AND (("Mental Disorders" OR "Mental Disorder*" OR "mental health problem*" OR "psychiatric disorder*" OR "mental health difficult*" OR "mental disease*" OR "mental illness*" OR "psychiatric disease*" OR "psychiatric illness*" OR "behavior disorder*" OR "behaviour disorder*" OR "behavioral disorder*" OR "behavioural disorder*" OR "behavior disorder*" OR "behaviour disorder*" OR "behavioral disorder*" OR "behavioural disorder*" OR "behavior problem*" OR "behaviour problem*" OR "behavioral problem*" OR "behavioural problem*" OR "internalizing disorder*" OR "externalizing disorder*" OR "thought disorder*" OR "anxiety disorder*" OR "eating disorder*" OR "PTSD" OR "posttraumatic stress disorder*" OR "post traumatic stress disorder*" OR "post-traumatic stress disorder*" OR "bipolar disorder*" OR "impulse control" OR "conductive disorder*" OR "disruptive disorder*" OR "oppositional defiant disorder*" OR ("autis*" AND "disorder*") OR "ADHD" OR "Attention Deficit Disorder*" OR "mood disorder*" OR "depression*" OR "depressive disorder*" OR "schizophreni*" OR "psychos*" OR "psychotic" OR "personality disorder*" OR "substance related disorder*" OR "substance-related disorder*" OR "substance disorder*" OR "alcohol disorder*" OR "alcohol abus*" OR ("Marijuana" AND "abus*") OR "Amphetamine-Related Disorder*" OR "Amphetamine Related Disorder*" OR ("XTC" AND "abus*") OR "drug abus*" OR "drug use disorder*"))) AND (("sever*" OR "intense" OR "suffering" OR "hardship*" OR "persisten*" OR "persisting" OR "enduring" OR "endures" OR "endure" OR "endured" OR "early onset" OR "lifetime" OR "life time" OR "life long" OR "lifelong") AND ("predict*" OR "risk" OR "riskfactor*" OR "preventi*" OR "risk" OR "risks" OR "Child Abuse" OR "Child Abuse" OR "maltreatment*" OR "maltreated" OR "Bullying" OR "bully*" OR "bullie*" OR "Self Concept" OR "self-esteem*" OR "trauma" OR "traumas" OR "psychotrauma*" OR "attach*" OR "Social Support" OR "social support*" OR "belongingness" OR "epistemic trust*" OR "social bind*" OR "hyper vigil*" OR "hypervigil*" OR "suicide" OR "suicid*" OR "treatment refusal" OR "biopsychosocial" OR "psychosocial" OR (("therapy" OR "treatment*" OR "therapeutics") AND ("resist*" OR "refus*")) OR ("suffer*" AND "intense") OR ("parent*" AND "abus*" AND ("acohol*" OR "drug" OR "drugs" OR "substance*")) OR ("parent*" AND ("behavior*" OR "behaviour*" OR "stress" OR "low income*")) OR "family history" OR "adopt*" OR "demograph*" OR "socioeconomic*" OR "socio-economic*" OR ("low" AND "educat*")) AND ("complex" OR "comorbidity" OR "comorbid*" OR "multimorbid*" OR "mulmorbid*" OR "multi-morbid*" OR ("multiple" AND ("disorder*" OR "illness*" OR "morbidit*")))))) OR (("child" OR "child" OR "children" OR "young adult" OR "adolescent" OR "schoolchild" OR "schoolchildren" OR "youngster" OR "youngsters" OR "boy" OR "boys" OR "girl" OR "girls" OR "Adolescent" OR "adolescent" OR "adolescents" OR "adolescence" OR "schoolage" OR "schoolboy" OR "schoolboys" OR "schoolgirl" OR "schoolgirls" OR "prepuber" OR "prepubers" OR "prepuberty" OR "puber" OR "pubers" OR "puberty" OR "teenager" OR "teenagers" OR "teens" OR "youth" OR "youths" OR "underaged" OR "under-aged" OR "Pediatrics" OR "Pediatric" OR "Pediatrics" OR "Paediatric" OR "Paediatrics") AND ("Mental Disorders" OR "Mental Disorder*" OR "mental health problem*" OR "psychiatric disorder*" OR "mental health difficult*" OR "mental disease*" OR "mental illness*" OR "psychiatric disease*" OR "psychiatric illness*" OR "behavior disorder*" OR "behaviour disorder*" OR "behavioral disorder*" OR "behavioural disorder*" OR "behavior disorder*" OR "behaviour disorder*" OR "behavioral disorder*" OR "behavioural disorder*" OR "behavior problem*" OR "behaviour problem*" OR "behavioral problem*" OR "behavioural problem*") AND ("severe" OR "suffering" OR "hardship*" OR "persisten*" OR "persisting" OR "enduring" OR "endures" OR "endure" OR "endured" OR "early onset" OR "lifetime" OR " life time" OR "life long" OR "lifelong") AND ("predict*" OR "riskfactor*" OR "preventi*" OR "risk" OR "risks" OR "Child Abuse" OR "maltreatment*" OR "maltreated" OR "bully*" OR "bullie*" OR "Self Concept" OR "self-esteem*" OR "trauma" OR "traumas" OR "psychotrauma*" OR "attach*" OR "social support*" OR "belongingness" OR "epistemic trust*" OR "social bind*" OR "hyper vigil*" OR "hypervigil*" OR "suicid*" OR "treatment refusal" OR "biopsychosocial" OR "psychosocial" OR (("therap*" OR "treatment*") AND ("resist*" OR "refus*")) OR ("suffer*" AND "intense") OR "parental abus*" OR ("parent*" AND "abus*" AND ("acohol*" OR "drug" OR "drugs" OR "substance*")) OR ("parent*" AND ("behavior*" OR "behaviour*" OR "stress" OR "low income*")) OR "family history" OR "adopt*" OR "demograph*" OR "socioeconomic*" OR "socio-economic*" OR ("low" AND "educat*")) AND ("complex" OR "comorbidity" OR "comorbid*" OR "multimorbid*" OR "mulmorbid*" OR "multi-morbid*" OR ("multiple" AND ("disorder*" OR "illness*" OR "morbidit*"))))
