# Supplementary material for: A systematic review exploring characteristics of youth with severe and enduring mental health problems (SEMHP)
Source: Eur Child Adolesc Psychiatry. 2023 Apr 24;33(5):1313–25. doi: 10.1007/s00787-023-02216-6 (PMC11098915; doi:10.1007/s00787-023-02216-6)
Supplement: Supplementary file 2 — Supplementary file2 (DOCX 81 KB) [file 787_2023_2216_MOESM2_ESM.docx]

**Appendix B. Study characteristics**

| Study number | Author (year) | Design  (method) | Respondents (size) | Study target group description | Age (participants/  target group) | Duration of mental health problems | Classifications | Quality study |
| --- | --- | --- | --- | --- | --- | --- | --- | --- |
| [57] | Wunderlich, Bronisch, and Wittchen (1998) | Cohort study (Questionnaires) | Youth + parents (n=3,021) | Youth with comorbid mental health problems with suicide attempts | 14-24y/o  (M=unknown) |  | Post-traumatic stress disorder, dysthymia, simple phobia, bipolar disorder, panic attack, social phobia, obsessive compulsive disorder, agoraphobia, illicit substance abuse/dependence, nicotine dependence, major depressive disorder, alcohol abuse/dependence, somatoform disorder, eating disorder | Medium |
| [63] | Becker-Weidman et al. (2009) | Cross-sectional analytical study (Diagnostic questionnaires) | Youth  (n=439) | Hopeless depressed youth | 12-17y/o  (M=14.6) |  | Mood disorders | Low |
| [45] | Peyre et al. (2017) | Cross-sectional analytical study (Diagnostic questionnaires) | Youth  (n=43,093) | Youth with comorbid mental health problems with attempt suicide <13y/o and 13-17y/o | 18-29y/o with suicide problems between 13-17y/o  (M=unknown) |  | Substance use disorder mood disorders, mania or hypomania, anxiety disorders, attention deficit hyperactivity disorder, pathological gambling, conduct disorder | High |
| [35] | Ciao, Accurso, Fitzsimmons-Craft, Lock, and Le Grange (2015) | Cohort study  (Surveys) | Youth + parents (n=121) | Youth with comorbid Anorexia Nervosa | 12-18y/o  (M=14.4) | 8.6 weeks | Anorexia Nervosa, anorexia Nervosa- bulimia, depression, obsessive compulsive disorder | Medium |
| [44] | Mueser and Taub (2008) | Case control study  (Interviews + diagnostic questionnaires | Youth + parents (n=69) | Youth with severe emotional disorders and PTSD who experienced trauma | 12-17y/o  (M=14.13) |  | Post-traumatic stress disorder, other anxiety disorder, mood disorder, conduct disorder, obsessive compulsive disorder, attention deficit hyperactivity disorder | Medium |
| [49] | Hoeve, McReynolds, and Wasserman (2015) | Cross-sectional study  (Diagnostic questionnaires) | Youth  (n=6,691) | Youth with comorbid internalizing and disruptive behavior disorder who experienced trauma | (M=15.4) |  | Internalizing disorder major depressive disorder, disruptive disorders, attention deficit hyperactivity disorder, alcohol abuse, alcohol dependence, marijuana abuse, marijuana dependence, other substance abuse, other substance dependence | High |
| [64] | Conway, Swendsen, Husky, He, and Merikangas (2016) | Cross-sectional study (Questionnaires) | Youth  (n=10,123) | Youth with prior lifetime mental disorders and both alcohol and illicit drug abuse, with or without dependence | 13-18y/o (M=15.2) |  | Any mood disorder, any phobia disorder, any anxiety disorder, any behavior disorder, any eating disorder, illicit drug use, regular alcohol use | High |
| [37] | Esposito, Spirito, Boergers, and Donaldson (2003) | Cross-sectional study  (Diagnostic questionnaires) | Youth  (n=121) | Youth attempted multiple suicide | 12-18y/o  (M=15) |  | Single attempters, multiple attempters, mood disorders, disruptive behavior disorder, alcohol use disorder, cannabis use disorder | High |
| [59] | McCarty et al. (2011) | Case control study  (Diagnostic questionnaire) | Youth  (n=198) | Youth with suicidal ideation with functional impairment | 13-17y/o  (M=15.2) |  | Substance use disorder | Medium |
| [60] | Merikangas et al. (2010) | Cross-sectional study (Questionnaires) | Youth  (n=10,123) | Youth with psychiatric comorbidity and substance use disorder | 13-18y/o  (M=15.2) |  | Mood disorders, anxiety disorders, behavior disorders, substance use disorders | High |
| [29] | Mekori et al. (2017) | Cohort study (Interviews + diagnostic questionnaires) | Youth  (n=88) | Comorbid hospitalized female youth with eating disorders | 15-18y/o  (M=15.98) | 28.8+/- 16 months | Anorexia nervosa, bulimia nervosa, eating disorder not otherwise specified, anxiety, depression, obsessive compulsive disorder | Medium |
| [40] | Bartoli et al. (2020) | Retrospective cohort study  (Diagnostic questionnaires) | Youth  (n=125) | Youth with Severe Mental Disorders | <25 y/o  (M=20.6) |  | Schizophrenia or other psychotic disorders, major depressive disorder, bipolar disorder, personality disorder, alcohol use disorders, substance use disorder | Low |
| [42] | King et al. (2006) | Case control study  (Diagnostic questionnaires) | Youth + parents (n=71) | Youth with comorbid major depressive disorder | 13-17y/o  (M=15.1) |  | Dysthymia, attention deficit hyperactivity disorder, obsessive compulsive disorder, behavior disorders, eating disorders, alcohol use disorder, cannabis use disorder | Medium |
| [58] | Georgiades, Paksarian, and Merikangas (2018) | Cross-sectional study  (Questionnaires) | Youth + parents (n=6,250) | Psychiatric youth with an immigration background | 13-18y/o  (M=15.26/ 15.07/ 15.00/ 14.87) |  | Behavior disorders, substance use disorder, depression, anxiety | High |
| [50] | Lehto-Salo, Närhi, Ahonen, and Marttunen (2009) | Cross-sectional study  (Diagnostic questionnaires) | Youth + parents (n=77) | Youth with comorbid OCD/ODD | 12-18y/o  (M=15.4) |  | Behavior disorders, schizophrenia, major depressive disorder, bipolar disorder, anxiety disorder, substance use disorder | Medium |
| [48] | Gattamorta, Mena, Ainsley, and Santisteban (2017) | Cross-sectional analytical study (Interviews + (diagnostic questionnaires) | Youth  (n=190) | Youth with mixed psychiatric disorder and substance use disorder | 14-17y/o  (M=15.95) |  | Behavior disorders, depression, anxiety, substance use disorder | High |
| [65] | Woody et al. (2019) | Review study | Youth + parents + CAP practitioners (n=43) | Youth with severe, persistent and complex mental illness | 12-25y/o  (M=unknown) |  | Mood disorders, obsessive compulsive disorder, substance use disorder, psychotic symptoms | Medium |
| [32] | Reiss et al. (2019) | Prospective cohort study (Questionnaires) | Youth + parents (n=2,111) | Youth with severe mental health problems | 7-17y/o & 9-19 y/o  (M =11.96/ 14.09) |  | Conduct disorder, attention deficit hyperactivity disorder | High |
| [39] | Swadi and Bobier (2003) | Retrospective cohort study  (Electronic files) | Youth  (n=62) | Youth with severe psychiatric illness and comorbidities with SUD | 16-18y/o  (M=16.35) |  | Mood disorder, major psychosis, anxiety disorder | Medium |
| [43] | Libby, Orton, Stover, and Riggs (2005) | Cross- sectional study  (Standardized diagnostic interviews) | Youth  (n=126) | Youth with major depressive disorder | 13-19y/o  (M=16.9) |  | Depression, substance use disorder | High |
| [38] | Scott, Lewis, and Marti (2019) | Cross-sectional analytical study (Diagnostic questionnaires) | Youth  (n=439) | Youth with comorbid depressive disorder | 12-17y/o  (M=14.6) |  | Major depressive disorder, anxiety disorder, severely disruptive disorder | High |
| [36] | Berona, Horwitz, Czyz, and King (2017) | Cohort study  (Diagnostic questionnaires) | Youth  (n=788) | Suicidal youth who are severely dysregulated | 13-17y/o  (M=15.6) |  | Anxious/depressed , withdrawn depressed with somatic complaints, with attention problems, with delinquency, with aggressive behavior | High |
| [62] | Wentz, Gillberg, Gillberg, and Råstam (2001) | Case control / Cohort study (Interviews) | Youth + parents (n=102) | Youth with comorbid anorexia nervosa | 16-24 y/o  (M=21/24.5/ 24.2) |  | Personality disorders, psychiatric disorders, depressive symptoms, obsessive-compulsive symptoms, autism spectrum disorder | High |
| [61] | Merikangas et al. (2011) | Cross-sectional study  (Questionnaires) | Youth + parents (n=6,483) | Youth with severe mental disorders | 13-18y/o  (M=15.9) |  | Any mood disorders, any anxiety disorders, behavior disorders, attention deficit hyperactivity disorder, substance use disorder, eating disorders | High |
| [46] | Rowe, Liddle, Greenbaum, and Henderson (2004) | Randomized controlled study  (Diagnostic questionnaires) | Youth + parents (n=182) | Youth with comorbid substance use disorder | 12-17y/o  (M=16.10/  15.31/15.28) |  | Substance abuse disorder, externalizing disorders, internalizing disorders | Medium |
| [28] | Goncalves, Marques, Cartaxo, and Santos (2020) | Case report  (Case presentation) | Mental health professional (n=1) | Youth with serious mental illness and multiple social risk factors | Teenager |  | x | Medium |
| [30] | Wittchen, Nelson, and Lachner (1998) | Cohort study  (Standardized diagnostic interviews) | Youth  (n=4,263) | Youth with mental disorders and psychosocial impairments | 14-24y/o  (M=unknown) |  | Any affective disorder, anxiety disorder, eating disorder, somatoform disorder | High |
| [56] | Nock et al. (2013) | Cross-sectional study  (Interviews + questionnaires) | Youth + parents  (n=6,483) | Adolescents with suicidal behavior | 13-18y/o  (M=unknown) |  | Fear/anger disorders, distress disorders, disruptive behaviors, substance abuse, substance disorders | Medium |
| [34] | Bühren et al. (2014) | Randomized controlled study  (Standardized diagnostic interviews) | Youth  (n=172) | Comorbid psychiatric female youth with first‐onset anorexia nervosa | 13-18y/o  (M=15.2) | 46.7 weeks | Affective disorders anxiety disorders, adjustment disorder, acute alcohol intoxication, attention deficit hyperactivity disorder | High |
| [27] | Bielas et al. (2016) | Case series  (Structured clinical interviews + self-reporting scale) | Youth  (n=130) | Mental disorders in detained male adolescent offenders with adverse childhood experiences | 13.8-19.5y/o  (M=17.4) |  | Post-traumatic stress disorder, depression, substance use disorder, disruptive disorder, attention deficit hyperactivity disorder | Medium |
| [51] | Zubrick et al. (2016) | Cross-sectional analytical study (Diagnostic questionnaires) | Youth + parents (n=8,963) | Youth with mental disorders and suicidal behavior | 12-17y/o  (M=unknown) |  | Social phobia, separation anxiety disorder, major depressive disorder, generalized anxiety disorder, conduct disorder, obsessive compulsive disorder, attention deficit hyperactivity disorder | Medium |
| [54] | Häberling et al. (2019) | Cross-sectional analytical study (Diagnostic questionnaires) | Youth  (n=126) | Youth with anxious major depressive disorder | 8-18y/o  (M=15.1) |  | Anxiety, major depressive disorder, attention deficit hyperactivity disorder psychosomatic complaints, aggression, gambling, trauma, mobbing, adjustment disorder, self-harm, suicidal behavior | High |
| [53] | Broersen, Frieswijk, Kroon Vermulst, and Creemers (2020) | Prospective cohort study (Questionnaires) | Youth  (n=199) | Young patients with persistent and complex care | 12-24y/o  (M=18.6) |  | Trauma, stressor-related disorders, mood disorder, autism, anxiety disorder, attention deficit hyperactivity disorder | Medium |
| [33] | Rice et al. (2014) | Qualitative study  (Focus groups) | Clinicians  (n=12) | Young people diagnosed with major depressive disorder | Clinicians (25-45 y/o) (M=34) of youth aged 15-25y/o |  | Depression, non-psychotic bipolar disorder | High |
| [47] | Chan, Dennis, and Funk (2008) | Cross-sectional study  (Interviews + diagnostic questionnaires) | Youth  (n=4,939) | Youth with comorbid internalizing and externalizing disorders and substance use disorder | 13-25y/o  (M=13.6/ 16.0/ 20.1) |  | Any substance use problems, alcohol use, amphetamine use, cannabis use, cocaine use, hallucinogen use, inhalant use, opioid use, sedative use, other drug use disorder-NOS, polysubstance dependence, internalizing problems, both internalizing and externalizing disorders | Medium |
| [55] | Lewinsohn, Rohde, and Selly (1994) | Cohort study (Interviews + diagnostic questionnaires | Youth  (n=1,709) | Youth with major psychiatric disorders (depression, anxiety, substance use, and disruptive behavior) and comorbid forms | 14-19y/o  (M=16.6) |  | Major depressive disorder, anxiety disorder, substance use disorder | Medium |
| [31] | T. Hirota, D. Paksarian, J-P. He, S. Inoue, E.K. Stapp, A. van Meter, & K.R. Merikangas (2022) | Cross-sectional study (Survey) | Parents (n=6,483) | Youth with severe lifetime disorders and/or comorbid disorders | Parents of 13-18 y/o (M=unknown) |  | Mood disorder, anxiety disorder, behavior disorders, substance use disorders, and eating disorders. | High |
| [41] | A. Gerdner & A. Hakansson (2022) | Cohort study (Diagnostic interviews) | Youth (n=387) | Youth with comorbid psychiatric disorders, including substance use disorder and gambling disorder | 18 y/o (M=unknown) |  | Depression, panic disorder, anxiety disorder, obsessive compulsive disorder, attention deficit hyperactivity disorder, gambling disorder, and psychotic disorder | Medium |
| [52] | K. Göbel, N. Ortelbach, C. Cohrdes, F. Baumgarten, A-K. Meyros, U. Ravens-Sieberer, & H. Scheithaur (2022) | Cohort study (Survey) | Youth + Parents (n=1,255) | Young people with internalizing, externalizing or co-occurrent mental health problems | 8-26 y/o (M=12.3 & M=18.5) |  | Attention deficit hyperactivity disorder, anxiety disorder, depression | High |
