# Supplementary material for: A systematic review exploring characteristics of youth with severe and enduring mental health problems (SEMHP)
Source: Eur Child Adolesc Psychiatry. 2023 Apr 24;33(5):1313–25. doi: 10.1007/s00787-023-02216-6 (PMC11098915; doi:10.1007/s00787-023-02216-6)
Supplement: Supplementary file 3 — Supplementary file3 (DOCX 27 KB) [file 787_2023_2216_MOESM3_ESM.docx]

**Appendix C. Summary of findings**

| Main theme | Theme (number of studies) | Size of evidence (study numbers) | Quality | Context | Consistency | Perspective | Areas of life | Strength of overall evidence^[[1]](#endnote-1)^ |
| --- | --- | --- | --- | --- | --- | --- | --- | --- |
| Descriptions and clinical associations |  |  |  |  |  |  |  |  |
| Descriptions | Severe (n=5) | 27, 28, 29  30, 31 | High quality: 4 Medium quality: 1 Low  quality: 0 | Mixed | Consistent | Mixed | Mixed | Very strong |
| Descriptions | Enduring (n=6) | 27, 29, 32  33, 34, 35 | High quality: 3 Medium quality: 3 Low  quality: 0 | Mixed | Inconsistent | Mixed | Mixed | Strong / Medium |
| Clinical associations with severe | Suicidality (n=7) | 29, 30, 34,  36, 37, 38,  39 | High quality: 5  Medium quality: 2 Low  quality: 0 | Mixed | Consistent | Mixed | Mixed | Strong |
| Clinical associations with severe and enduring | Comorbidity (n=4) | 30, 34, 40,  41 | High quality: 2 Medium quality: 2 Low  quality: 0 | Mixed | Consistent | Specific | Mixed | Medium |
| Contributing factors |  |  |  |  |  |  |  |  |
| Biological | Heredity (n=7) | 27, 29, 42,  43, 44, 45  46 | High quality: 1 Medium quality: 6 Low  quality: 0 | Mixed | Inconsistent | Mixed | Mixed | Medium |
| Biological | Age (n=9) | 30, 32, 46,  47, 48, 49  50, 51, 52 | High quality: 5 Medium quality: 4 Low  quality: 0 | Mixed | Mixed | Mixed | Mixed | Strong |
| Biological | Gender (n=16) | 27, 30, 38,  39, 41, 43,  45, 46, 48,  51, 52, 53,  54, 55, 56,  57 | High quality: 6 Medium quality: 10 Low  quality: 0 | Mixed | Mixed | Mixed | Mixed | Strong/ Medium |
| Psychological | Trauma (n=12) | 27, 28, 29,  42, 43, 44,  45, 47, 49,  50, 53, 57 | High quality: 3 Medium quality: 9 Low  quality: 0 | Mixed | Consistent | Mixed | Mixed | Very strong/ Strong |
| Sociological | Socio-economic (n=21) | 27, 28, 30,  32, 36, 37,  38, 42, 44,  46, 48, 50,  51, 53, 56,  57, 58, 59,  60, 61, 62 | High quality: 10 Medium quality: 11 Low  quality: 0 | Mixed | Inconsistent | Mixed | Mixed | Strong |
| Sociological | Family functioning (n=14) | 28, 31, 35,  39, 43, 44,  46, 48, 51,  55, 56, 57,  60, 61 | High quality: 4 Medium quality: 9 Low  quality: 1 | Mixed | Mixed | Mixed | Mixed | Strong/ Medium |
| Sociological | Peer support (n=5) | 31, 39, 45,  53, 63 | High quality: 2 Medium quality: 2 Low  quality: 1 | Mixed | Inconsistent | Mixed | Mixed | Medium |
| Sociological | Ethnical (n=6) | 27, 36, 45,  48, 58, 60 | High quality: 5 Medium quality: 1 Low  quality: 0 | Mixed | Mixed | Mixed | Mixed | Very strong/  Strong |
| Impact |  |  |  |  |  |  |  |  |
| Impact- Youth | Academic (n=8) | 28, 29, 30,  34, 53, 54,  55, 60 | High quality: 4 Medium quality: 4 Low  quality: 0 | Mixed | Consistent | Mixed | Mixed | Very strong/  Strong |
| Impact - Youth | Psychosocial (n=11) | 28, 29, 30,  34, 44, 49,  53, 54, 55,  57, 60 | High quality: 4 Medium quality: 7 Low  quality: 0 | Mixed | Consistent | Mixed | Mixed | Very strong / Strong |
| Impact- Youth | Hopelessness (n=5) | 33, 36, 37,  38, 63 | High quality: 3 Medium quality: 1  Low  quality: 1 | Mixed | Inconsistent | Specific | Mixed | Medium |
| Impact- Youth | Suicide attempts (n=15) | 27, 29, 34,  36, 37, 39,  44, 45, 47,  50, 51, 54,  55, 56, 57 | High quality: 4  Medium quality: 11  Low  quality: 0 | Mixed | Mixed | Mixed | Mixed | Strong |
| Impact- Youth | Substance abuse (n=7) | 27, 28, 36,  39, 49, 64,  65 | High quality: 2 Medium quality: 5 Low  quality: 0 | Mixed | Inconsistent | Mixed | Mixed | Strong/ Medium |
| Impact- Youth | Criminal behavior (n=7) | 27, 38, 44,  47, 49, 53,  65 | High quality: 2 Medium quality: 5 Low  quality: 0 | Mixed | Consistent | Mixed | Mixed | Very strong/ Strong |
| Impact- Societal | Cost (n=1) | 28 | High quality: 0 Medium quality: 1 Low  quality: 0 | Specific | Consistent | Specific | Specific | Limited/ No evidence |
| Impact- Societal | Policies (n=2) | 28, 53 | High quality: 0 Medium quality: 2 Low  quality: 0 | Mixed | Consistent | Mixed | Specific | Medium |

1. Weighting of related studies: study #30 & study #57 count as 1; study #38 & study #63 count as 1 and; study #31 & study #60 & study #61 & study #64

   count as 1. [↑](#endnote-ref-1)
